# Efficacy of Internet-Based Therapies for Tinnitus: A Systematic Review and Meta-Analysis of Randomized Controlled Trials

**DOI:** 10.3390/jpm14080813

**Published:** 2024-07-31

**Authors:** Egidio Sia, Giancarlo Tirelli, Annalisa Gatto, Chiara Angela Mineo, Kaveri Curlin, Mehdi Abouzari

**Affiliations:** 1 Department of Medical, Surgical and Health Sciences, Section of Otolaryngology, University of Trieste, 34149 Trieste, Italy; 2 Division of Neurotology and Skull Base Surgery, Department of Otolaryngology-Head and Neck Surgery, University of California, Irvine, CA 92697, USA

**Keywords:** tinnitus, internet-based treatment, Tinnitus Functional Index (TFI), Tinnitus Handicap Inventory (THI), Tinnitus Reactions Questionnaire (TRQ)

## Abstract

Background: Tinnitus presents a major public health challenge, impacting quality of life. With conventional therapies being often time-consuming and costly, interest in Internet-based treatments, such as auditory treatments and Internet-based cognitive behavioral therapy, has grown due to their improved patient adherence. This meta-analysis aims to review existing scientific literature to assess the effectiveness of Internet-based therapies (IBTs) in treating tinnitus. Methods: Studies up to February 2024 using the Tinnitus Functional Index (TFI), Tinnitus Handicap Inventory (THI), or Tinnitus Reactions Questionnaire (TRQ) to monitor tinnitus before and after IBTs were searched in PubMed, Google Scholar, Web of Science, and the Cochrane Central Register of Controlled Trials. Variation of the score with time was analyzed and a comparison was made with non-IBT studies. Treatment effects were analyzed using Cohen’s d model. Results: A total of 14 articles were considered, with a total of 1574 patients. Significant improvements in questionnaire scores were noted post-treatment. In the IBT group, THI and TFI decreased by 17.97 and 24.56 points, respectively (Cohen’s d THI: 0.85; TFI: 0.80). In the control group, THI and TFI decreased by 13.7 and 4.25 points, respectively (Cohen’s d THI: 0.55; TFI: 0.10). Conclusions: Internet-based therapies showed reliable effectiveness, possibly due to improved patient compliance, accessibility, cost-effectiveness, and customization.

## 1. Introduction

Tinnitus, colloquially referred to as “ringing in the ears”, is “an auditory sensation without an external sound stimulation or meaning, which can be lived as an unpleasant experience, possibly impacting quality of life” [1]. Over the past 20–30 years, it has emerged as a serious public health issue, affecting 760 million people worldwide [2,3]. Tinnitus is a symptom, not a disease, and although it is often associated with curable and non-critical conditions, it impacts quality of life, partly due to inherent challenges in its treatment [4]. In fact, it often manifests without any objective signs, and due to inadequate understanding of its pathology, it can be a symptom of various disorders such as otological, orofacial, cardiovascular, and neurological diseases [5]. Tinnitus may originate from cochlear abnormalities or hearing loss and is then maintained by neural changes in the central auditory system, with altered neuronal activity [5]. Arterial and arteriovenous pulsatile tinnitus could result from arterial stenosis, skull base anatomical variants, or vascular skull base tumors, while somatic inputs such as temporomandibular disorders can influence tinnitus perception in a condition known as “somatosensory tinnitus” [6,7]. As a neurological disorder, it is known that tinnitus may be associated with migraine phenomena, as both are similarly elusive in etiology but possibly share a pathophysiology linked by the central nervous system [8]. The absence of an objective measure for tinnitus has resulted in self-report questionnaires being the preferred method to evaluate tinnitus symptoms and quantify the degree to which quality of life is affected negatively [9]. There are several tinnitus questionnaires available, with widely used ones including the Tinnitus Reaction Questionnaire (TRQ) [10], Tinnitus Handicap Inventory (THI) [11], and Tinnitus Functional Index (TFI) [12]. The THI is a 25-item survey with three subscales: functional (12 items), emotional (eight items), and catastrophic response (five items), and is a robust measure of tinnitus impact on everyday life. The TFI, also a 25-item index, scales tinnitus severity, identifies domains affecting it and measures treatment-related changes, with scores ranging from 0 to 100, categorized into five severity levels from ‘not a problem’ to ‘very big problem’. The TRQ consists of 26 items rated from 0 to 4, summed into a total score from 0 to 104, and describes the distress associated with tinnitus. In all three questionnaires, higher scores correspond to greater severity of tinnitus. After assessing the severity of tinnitus and its impact on quality of life, treatment in these patients should focus on addressing the underlying or concurrent disorder (if present) and implementing specific treatments aimed at reducing the severity of the tinnitus. Treatments for managing tinnitus include psychological interventions such as counseling and psychoeducation, auditory stimulation such as sound therapy, and, when necessary, interventions to reduce distress such as relaxation therapy [2,13,14]. Based on the possibility that tinnitus is linked to high spontaneous neuronal activity, brain stimulation therapies, such as transcranial magnetic stimulation, transcranial direct current stimulation, and deep brain stimulation, represent valid treatment options for these patients [5,15,16,17]. Furthermore, in recent years, there has been a growing interest in Internet-based therapies (IBTs). These approaches hold promise in overcoming some of the limitations associated with conventional therapies, ensuring accessibility, cost reduction, and consequently enhancing patient compliance. They include questionnaires, auditory treatments, internet-based cognitive behavioral therapy (iCBT), and games present in different operating systems for tinnitus monitoring and management [4].

The purpose of this study was to review peer-reviewed publications on the effectiveness of internet-based therapies for tinnitus to analyze the efficacy of these treatments. The primary aim was to assess tinnitus improvement resulting from IBT by examining how patient questionnaire scores (THI, TFI, and TRQ) varied over time, reported as medians and with a 95% confidence interval. The secondary objective was to compare outcomes between patients receiving IBT (case group) and non-IBT patients (control group treatment, CGT). This meta-analysis intentionally selected studies concerning IBTs that vary significantly from one another, both in terms of delivery methods (e.g., smartphone-based, web-based, computer-based) and types of therapy (e.g., cognitive-behavioral therapy, music therapy). This approach has allowed us to include a larger number of studies and to analyze the overall effectiveness of IBT compared to face-to-face therapies.

## 2. Materials and Methods

### 2.1. Search Strategy

This manuscript is based on the PRISMA guidelines. PROSPERO registration was completed (ID 565308). The databases PubMed, Google Scholar, Web of Science, and the Cochrane Central Register of Controlled Trials were searched up to February 2024. The bibliographic research was conducted by a health sciences librarian. The search syntax was adapted for each database to account for variation in thesaurus terms/controlled vocabulary across databases. Keywords were used to identify paper sources that included the following: (1) the condition being studied (“tinnitus”); (2) the proposed intervention (“therapy” OR “intervention” OR “treatment”); (3) the method of intervention delivery (“mobile” OR “internet” OR “in-person”). Covidence software (https://www.covidence.org/ accessed on 1 July 2024) was used for the research and to deduplicate results. Exact search terms used in each database are presented in Appendix A.

### 2.2. Study Selection 

Only studies that quantified the intensity of tinnitus using the TFI, THI, and TRQ were considered. Only full text articles were included. The PICO framework was employed to clearly define study eligibility, as follows: (P) patients with tinnitus; (I) Internet-based therapies (including mobile, internet, and in-person); (C) control patients; (O) variation of tinnitus intensity based on the chosen questionnaires.

The exclusion criteria were as follows: (1) reviews, editorials, and non-full-text articles; (2) non-English language studies; (3) studies that evaluated a therapeutic period shorter than 6 weeks; (4) studies containing aggregated and non-extractable data, or duplicated data from previously published work.

### 2.3. Data Extraction 

An electronic data-collection form was used to collect the data. The following information were collected: (1) study information: author, year of publication, country of the cohort, type of study; (2) Internet-based therapies: modality, type of treatment, duration of treatment, type of questionnaire, number of patients pre- and post-treatment, pre- and post-treatment median and standard deviation across various questionnaires; (3) follow-up: number of patients, median and standard deviation across various questionnaires at 2, 3, 6, and 12 months; (4) control group: treatment type, number of patients, median and standard deviation across different questionnaires before and after treatment.

### 2.4. Statistical Analysis 

The median scores across different questionnaires, along with the number of patients and standard deviations for both cases and controls, were extracted from the studies. A 95% confidence interval (CI) was calculated for each questionnaire, assessing scores pre-treatment and at the end of treatment. Additionally, scores harvested 12 months after the beginning of therapy were considered in the cases group. Calculations were performed using random effects models by DerSimonian and Laird, employing a weighted average approach that assigned each study a weight proportional to its precision and incorporated both within- and between-study variability [18]. The results of the meta-analysis were graphically presented using forest plots, which included summary estimates and a corresponding 95% CI. Differences in the effect size among the cases over time and disparities in outcomes between cases and controls were assessed using Cohen’s d model. All statistical analysis were calculated using RStudio Desktop, version 2023.09.0+463.

## 3. Results

### 3.1. Search Results and Study Selection

**Figure 1:**
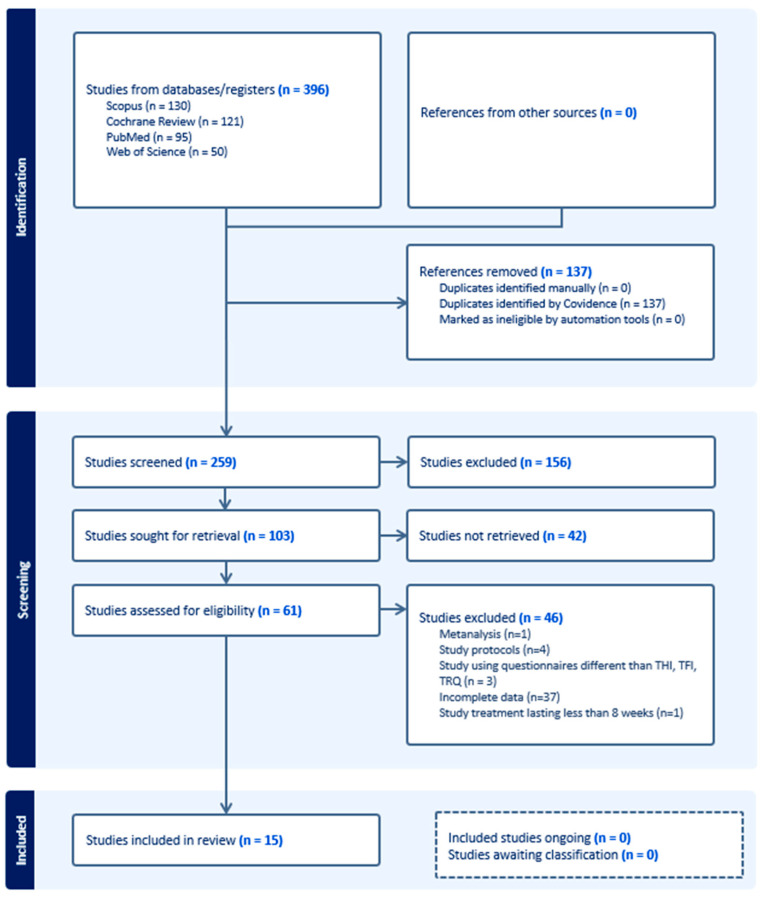
Selection of articles according to the PRISMA statement flow diagram.

Once the duplicates were eliminated, 259 items were screened, leading to the exclusion of 156 articles based on the title. The full text of the remaining 103 articles was further reviewed, and 15 articles met the inclusion criteria for the meta-analysis, as depicted in Figure 1. Data describing the characteristics of the included studies are reported in the Table presented in Appendix B.

The work of Searchfield and colleagues [19] was excluded from the statistical analysis due to the presentation of its results, which were not compatible with our data collection and statistical analysis procedures. The included articles [20,21,22,23,24,25,26,27,28,29,30,31,32,33] involved 1574 patients. Among the selected articles, nine studies were randomized control trials (RCTs), two were pilot trials, one was a repeated measure design, one an effectiveness trial, one a preliminary study, and one a single group open trial. Two of the articles [23,28] described RCTs where patients were monitored using two questionnaires; for this reason, they were considered separately for our statistical analysis. Two other studies [25,26] were three-arm RCTs, with, respectively, two case groups and two control groups. For this reason, they were considered as different studies for our statistical analysis; thus, they were counted as four separated studies. 

### 3.2. Pre- and Post-Internet-Based Treatment (IBT) Differences

**Figure 2:**
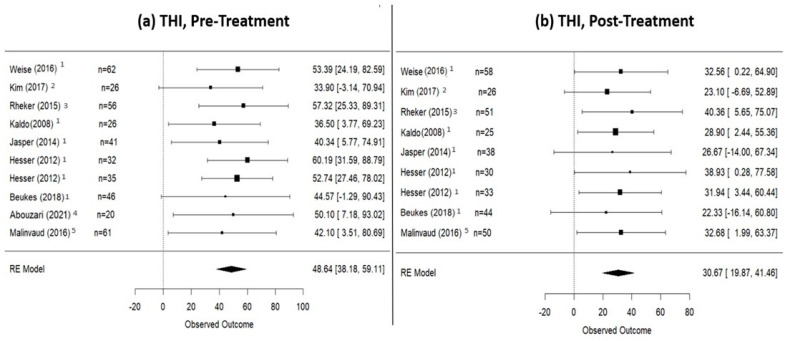
THI scores in patients who underwent internet-based therapies. (**a**) Pre-treatment scores; (**b**) post-treatment scores. This figure shows a THI decrease of 17.97 points (from 48.64 pre-treatment to 30.67 post-treatment). 1: iCBT studies; 2: a sound therapy study; 3: an iCBT + therapist study; 4: an iCBT + sound therapy study; 5: a virtual reality study [21,23,26,27,29,30,31,32,34].

**Figure 3:**
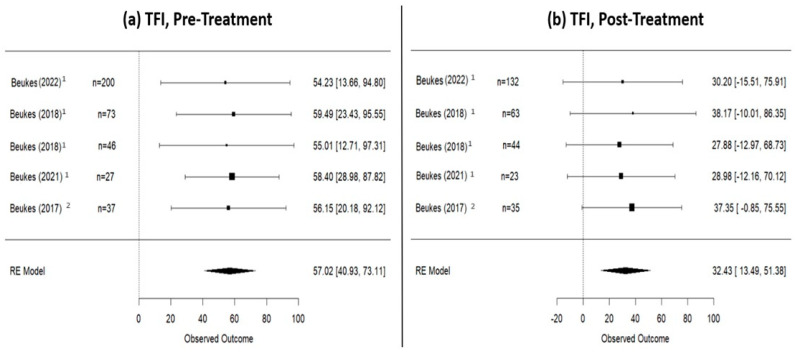
TFI scores in patients who underwent internet-based therapies. (**a**) Pre-treatment scores; (**b**) post-treatment scores. This figure shows a TFI decrease of 24.59 points (from 57.02 pre-treatment to 32.43 post-treatment). 1: iCBT studies; 2: an iCBT + therapist study [22,23,24,25,33].

**Figure 4:**
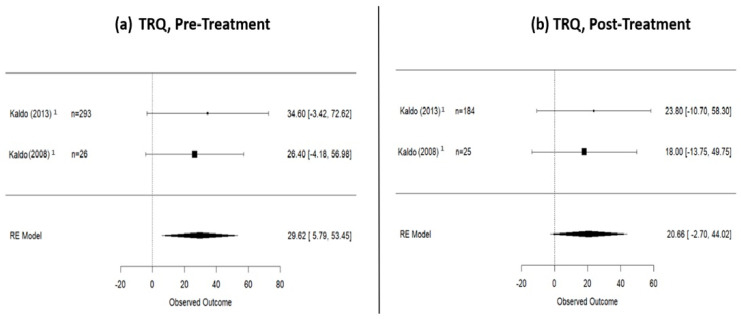
TRQ scores in patients who underwent internet-based therapies. (**a**) Pre-treatment scores; (**b**) post-treatment scores. This figure shows a TRQ decrease of 8.96 points (from 29.62 pre-treatment to 20.66 post-treatment). 1: iCBT studies [28,29].

A total of 987 patients underwent IBT between 8 and 12 weeks. In particular, 800 participants underwent iCBT treatment (internet-delivered cognitive behavioral therapy) [21,22,23,24,25,26,27,28,31], 93 iCBT with therapist guidance [30,32], 26 were subjected to music therapy [29], 35 participated in iACT (internet-delivered acceptance and commitment therapy) [25], 20 engaged in a combined treatment approach of iCBT and music therapy [20], and 61 underwent virtual reality treatment [33]. In terms of outcomes, before commencing the treatment, 405 patients were assessed with the THI questionnaire, 383 with the TFI questionnaire, and 319 with the TRQ. At the conclusion of the treatment, a total of 812 patients had their tinnitus assessed, with 355, 297, and 209 patients, respectively, using the THI, TFI, and TRQ questionnaires. According to the random effects model calculation (Figure 2), the mean THI score in patients who underwent Internet-based therapy was 48.64 (95% CI: 38.18–59.11) at pre-treatment, decreased to 30.67 (95% CI: 19.87–41.46) at post-treatment, and slightly increased to 36.44 (95% CI: 15.34–57.53) at the 1-year follow-up. Data regarding TFI score are presented in Figure 3: at pre-treatment, the TFI score was 57.02 (95% CI: 40.93–73.11), which decreased to 32.43 (95% CI: 13.49–51.38) at post-treatment and remained relatively stable at the 1-year follow-up [33.43 (95% CI: −0.77–67.62)]. Finally, the mean TRQ score (Figure 4) at pre-treatment was 29.62 (95% CI: 5.79–53.45), and at post-treatment, it was 20.66 (95% CI: −2.70–44.02). Data on tinnitus status at the 1-year follow-up, assessed using THI and TFI questionnaires, are provided in Appendix C.

### 3.3. Control Group Treatment (CGT)

**Figure 5:**
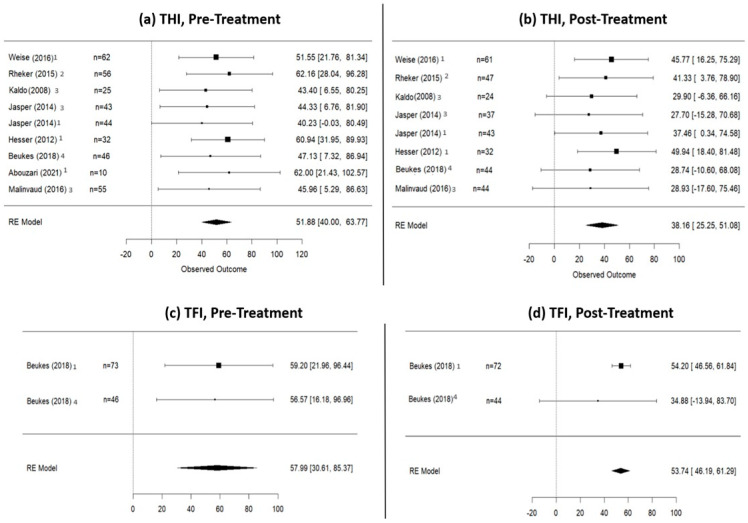
THI and TFI scores of the control group treatment (CGT, patients who did not undergo internet-based therapies). This figure shows a THI decrease of 13.72 points and a TFI decrease of 4.25 in the CGT group. (**a**) Pre-treatment THI scores; (**b**) post-treatment THI scores; (**c**) pre-treatment TFI scores; (**d**) post-treatment TFI scores. 1: studies with DF as control group treatment; 2: a study with iCBT as control group treatment; 3: studies with CBT as control group treatment; 4: a study with F2F as control group treatment [21,23,24,26,27,29,31,32,34].

The total number of patients included in the CGT was 494. Specifically, 221 patients participated in a discussion forum (DF), 179 underwent cognitive-behavioral therapy (CBT), 46 received face-to-face therapy (F2F), and 48 underwent music therapy. Before commencing the treatment, 373 patients were assessed using THI, 119 using TFI, and 25 using TRQ. At the conclusion of treatment, the total number of controls was 414, with 332, 116, and 24 using THI, TFI, and TRQ, respectively, to assess tinnitus improvement. According to the random effects model calculation, the mean THI score in the control group was 51.88 (95% CI: 40.00–63.77) at pre-treatment, notably decreasing to 38.16 (95% CI: 25.25–51.80) at post-treatment. The mean TFI score at pre-treatment was 57.99 (95% CI: 30.61–85.37), decreasing to 53.74 (95% CI: 46.19–61.29) at post-treatment. Due to having only one study with TRQ scores in the control group, a forest plot for TRQ values in the control group was not created [28]. Data are shown in Figure 5. 

### 3.4. Size Effect Analysis

In order to measure the effect size of IBT, calculations were made according to a Cohen’s d model to highlight the standardized difference between the means of two groups. Cohen’s d values should be interpreted as follows: small effect (around 0.2), medium effect (around 0.5), and large effect (around 0.8 or higher). Cohen’s d was calculated based on the pre- vs. post-IBT variation in the scores of THI and TFI, aiming to highlight the effect of IBT on tinnitus (Table 1). The Cohen’s d for THI and TFI was 0.85 and 0.71, respectively, indicating a high effect of the treatment on THI and a moderate–high effect on TFI. Cohen’s d for TRQ variation was 0.19, indicating a low effect of IBT.

Cohen’s d was also calculated for patients who did not receive IBT (case control group, CGT), resulting in 0.55 for THI variation and 0.11 for TFI. This indicates a moderate effect on THI score and almost no effect on TFI. Cohen’s d calculation was not performed in the CGT due to the limited sample size. Cohen’s d calculation was also used to examine potential baseline disparities between the control and case cohorts, aiming to elucidate the impact of IBT. Among patients monitored using the THI, Cohen’s d between cases and control was 0.15 at pre-treatment, while for those monitored with TFI, it was 0.03. These data show no baseline differences. Cohen’s d calculation among the IBT group and CGT are shown in Table 1, demonstrating a difference in outcomes among the groups at the end of treatment, which is low-to-moderate for THI and moderate for TFI.

## 4. Discussion

This systematic review and meta-analysis aimed to investigate the efficacy of internet-based therapies for patients with tinnitus. The management of tinnitus primarily involves identifying and treating any underlying organic causes. However, in most cases, the origin of tinnitus remains unknown, necessitating a focus on reducing the intensity of the symptom and the associated distress. For acute tinnitus, particularly when linked to sudden hearing loss, current guidelines recommend systemic or intratympanic corticosteroid therapy [34]. In the case of chronic tinnitus without an identifiable cause, various treatment options exist, though the supporting evidence is often limited. The clinical efficacy and safety of repetitive transcranial magnetic stimulation (rTMS) in chronic tinnitus have been studied, but results are frequently divergent and sometimes contradictory, highlighting the need for further research in this area [2]. Sound therapy is another treatment often considered, since there have been no associated adverse effects, even though evidence for sound therapy as an effective solo treatment is inconclusive [2,35]. CBT stands out as the only treatment with moderate- to high-quality evidence supporting its efficacy [2]. However, CBT also presents some limitations associated with low patient compliance. For this reason, therapies administered via the internet have gain interest. Our results revealed a significant improvement in tinnitus severity and quality of life among patients who underwent internet-based therapies compared to treatments such as cognitive behavioral therapy, discussion forum, and face-to-face therapy. 

Given the challenges in objectifying symptoms like tinnitus, THI, TFI, and TRQ questionnaires were used to assess both the subjectivity of the symptom and the efficacy of the treatment. More precisely, TRQ focuses on analyzing psychological distress, THI describes the impact of tinnitus on daily living, while TFI is validated to assess both the severity of tinnitus and measure treatment-related changes [9]. Our data highlighted that IBT positively impacted both THI and TFI (Cohen’s d pre- vs. post-IBT was, respectively, 0.85 and 0.71). This indicates that IBT had a high positive impact on everyday life, as reflected in the decrease in THI scores. Furthermore, the decrease in TFI scores suggests a moderate to high efficacy of IBT in treating symptoms of tinnitus. On the other hand, TRQ showed a low decrease in scores, which could be attributed to the fact that only two studies using TRQ were considered for the analysis. When available, data on tinnitus status at the 1-year follow-up were considered to determine the long-term efficacy of IBT. Compared to the end of treatment, at the 1-year follow-up, TFI increased by one point, and THI increased by 5.77 points. This slight increase in scores at the 1-year follow-up compared to those immediately after therapy could be explained by a greater short-term effect compared to the long term. Overall, the total case population sustained improvements over time compared to the beginning of therapy, albeit with a slight decline compared to the immediate post-treatment period.

Finally, a few more considerations can be made when comparing the IBT group and CGT. Considering that TFI and THI scores were almost overlapping pre-treatment among cases and controls (Cohen’s d lower than 0.2), there is a difference in the improvement of THI and TFI scores after treatment, with better results in the IBT group. Specifically, when comparing the IBT group with CGT, at the end of the treatment, the variations in THI and TFI describe a low–moderate difference in the subjective perception of tinnitus and a moderate difference in the efficacy of the treatment, respectively, highlighting better outcomes in the IBT group. These results could be attributed to better patient compliance resulting from the accessibility, cost-effectiveness, and customization of IBT.

While the number of available reviews is limited, our study aligns with existing literature, showing promise for future treatments. For instance, Beukes et al. [32] conducted a review specifically on internet-delivered cognitive behavioral therapy for tinnitus patients, finding a significant preference for Internet-based interventions over both inactive and active controls. Another systematic review by Demoen et al. [36] demonstrated low to moderate evidence of reduced tinnitus severity and distress in patients treated with telerehabilitation. However, high dropout rates due to factors like lack of time, engagement, motivation, and patient openness were noted, leading to concerns about bias and certainty levels. A slightly different conclusion was drawn from the systematic review conducted by Nagaraj et al. [4], which focused on internet- and smartphone-based applications for treating tinnitus. Their analysis revealed a comparable improvement in both traditional and internet-delivered forms of tinnitus treatment, highlighting their effectiveness. However, unlike previous studies, Nagaraj and colleagues did not find a significant difference in treatment outcomes for patients undergoing internet-delivered therapies than conventional ones. Moreover, various causes of tinnitus are described in the literature [5,37]. Some of these causes could be transient, a factor that should be considered when analyzing data regarding tinnitus. However, even if patients who would have naturally improved or recovered “on their own” were included in this analysis, our findings still demonstrate a better outcome in patients undergoing IBT compared to the CGT. 

IBTs hold promise in overcoming some of the limitations associated with conventional therapies, ensuring accessibility, cost reduction, and consequently enhancing patient compliance. More precisely, from a practical and economic perspective, the cost of management is a significant consideration for many patients with tinnitus. In countries with insurance-based healthcare systems, tinnitus management is often not covered by many insurance companies. Thus, the accessibility of internet-based therapies represents an important advancement in providing a non-invasive, supportive, and more affordable method of tinnitus management. Furthermore, given the easy accessibility associated with IBTs, these technologies could achieve widespread adoption among patients. This widespread use has the potential to significantly improve the quality of life for a large number of individuals, ultimately resulting in a more productive and functional role for patients in society.

A few considerations should be made about the limitations of this meta-analysis. First, the heterogeneity of treatment observed within both the patient and control groups presents a challenge. The meta-analysis intentionally did not focus on the comparison of a specific internet-based therapy with its face-to-face counterpart but rather sought to examine the overall efficacy of IBTs without focusing on specific modalities. The goal of this meta-analysis was indeed to assess the efficacy and reach of digital interventions as a whole. This certainly represents a limitation, but at the same time it could be considered as a direction for future studies. Despite the utilization of standardized questionnaires, the subjective nature of tinnitus symptoms and individual variations in response to it represent an element that is not easily standardizable into a meta-analysis. Furthermore, the small sample size hindered our ability to conduct uniform statistical analyses across all the groups of patients monitored with different questionnaires. This limitation underscores the need for larger and more diverse datasets to provide more robust conclusions in future studies. This approach would help identify the most suitable therapy to improve the quality of life of patients with tinnitus. Finally, exploring the variables that influence patient compliance and the adoption of IBTs could provide further insights into how to optimize these therapies for greater economic and therapeutic impact.

## 5. Conclusions

Despite improvements observed in patients undergoing therapies such as CBT, discussion forum, and face-to-face therapy, internet-based treatments have demonstrated reliable effectiveness in significantly reducing THI and TFI scores. This suggests that internet-based therapies offer a promising avenue for enhancing tinnitus management strategies, ensuring accessibility, reducing costs, and consequently improving patient compliance.

## Figures and Tables

**Table 1:** Cohen’s d calculation among different groups.

Groups Compared	Questionnaire Used	Cohen’s d Calculation
Pre- vs. post-IBT	THI	0.8460
Pre- vs. post-IBT	TFI	0.7068
Pre- vs. post-IBT	TRQ	0.1895
Pre- vs. post-CGT	THI	0.5541
Pre- vs. post-CGT	TFI	0.1052
Pre-CGT vs. pre-IBT	THI	0.1451
Pre-CGT vs. pre-IBT	TFI	0.02505
Post-CGT vs. post-IBT	THI	0.3156
Post-CGT vs. post-IBT	TFI	0.6432

Cohen’s d was calculated based on the THI, TFI, and TRQ scores at pre-IBT vs. post-IBT and at pre-CGT vs. post-CGT to show the effect size of the treatment on tinnitus. Cohen’s d was also calculated for the THI and TFI scores at pre-CBT vs. pre-IBT and at post-CBT vs. post-IBT to highlight any potential baseline disparities between the control and case cohorts. A Cohen’s d of 0.2 indicates a small effect size, 0.5 a medium effect size, and 0.8 a large effect size. IBT: Internet-based treatment; THI: Tinnitus Handicap Inventory; TFI: Tinnitus Functional Index; TRQ: Tinnitus Reactions Questionnaire; CGT: control group treatment.

**Figure A1:**
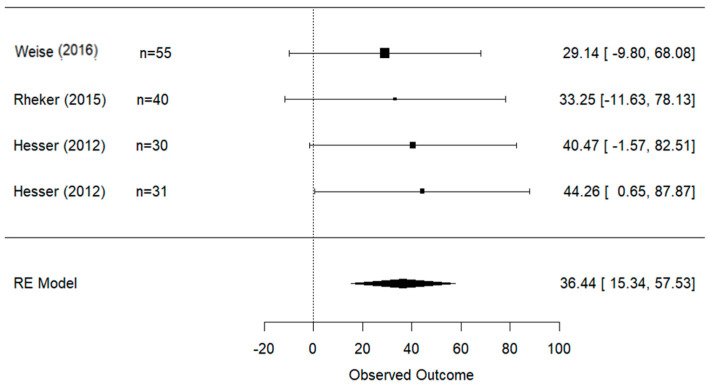
THI at 12-month follow-up in Internet-based treatment group [26,31,32].

**Figure A2:**
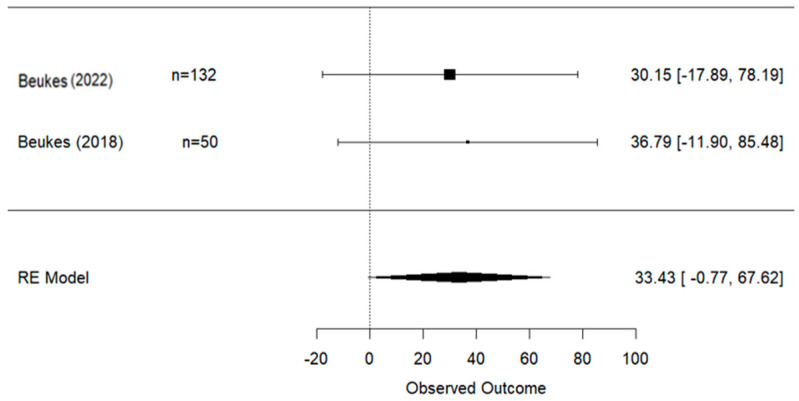
TFI at 12-month follow-up in in Internet-based treatment group [22,23].

## Data Availability

The data that support the findings of this study are available from the corresponding authors upon reasonable request.

## Appendix A

List of exact search terms used in each database.

PubMed Search. Search: (((Tinnitus[mh] OR Tinnitus[tiab]) AND (Therapy/Narrow[filter])) OR ((Tinnitus[mh] OR Tinnitus[tiab]) AND (Random*[tiab] OR RCT[tiab]))) AND (Smartphone OR &quot;smart phone*&quot; OR handheld computer OR Internet OR Mobile Device OR Tablet Computer OR Mobile Applications OR mobile app OR Telemedicine OR Online OR Virtual Medicine OR Telehealth OR eHealth OR Telecare OR Mobile Health OR mHealth OR virtualHealth OR &quot;Virtual Health&quot; OR virtual visit* OR Distance Counseling OR Telepathology OR Telerehabilitation OR E-therapy OR E-Counseling OR Virtual Healthcare)

Scopus Search. Tinnitus AND (Smartphone OR &quot;smart phone*&quot; OR “handheld computer” OR Internet OR “Mobile Device” OR Tablet OR “Mobile Application*” OR “mobile app” OR Telemedicine OR Online OR Virtual* OR Telehealth OR eHealth OR Telecare OR “Mobile Health” OR mHealth OR “Distance Counseling” OR Telepathology OR Telerehabilitation OR “E therapy” OR “E Counseling”)

Cochrane CENTRAL. 121 Trials matching tinnitus in Title Abstract Keyword AND (Smartphone OR &quot;smart phone&quot; OR handheld computer OR Internet OR Mobile Device OR Tablet Computer OR Mobile Applications OR mobile app OR Telemedicine OR Online OR Virtual Medicine OR Telehealth OR eHealth OR Telecare OR Mobile Health OR mHealth OR virtualHealth OR &quot;Virtual Health&quot; OR virtual visit* OR Distance Counseling OR Telepathology OR Telerehabilitation OR E-therapy OR E-Counseling OR Virtual Healthcare) in Title Abstract Keyword–(Word variations have been searched)

Web of Science Core Collection. Tinnitus AND (Smartphone OR &quot;smart phone*&quot; OR “handheld computer” OR Internet OR “Mobile Device” OR Tablet OR “Mobile Application*” OR “mobile app” OR Telemedicine OR Online OR Virtual* OR Telehealth OR eHealth OR Telecare OR “Mobile Health” OR mHealth OR “Distance Counseling” OR Telepathology OR Telerehabilitation OR “E therapy” OR “E Counseling”) (All Fields) and Random* OR RCT (All Fields) and Article (Document Types)

## Appendix B

**Table A1:** Characteristics of the included studies.

Author	Year of Publication	Country of Cohort	Total Number of Patients	Number of Case Group Patients	Modality of Treatment	Type of Treatment in Case Groups	Type of Treatment in Control Groups (if RCT)	Time of Treatment (Weeks)	Questionnaire Used
Weise	2016	Germany	124	62	PC	iCBT	DF	8	THI
Kim	2017	Korea	26	26	Smartphone	Music		12	THI
Rheker	2015	Germany	112	56	Person	iCBT + therapist	iCBT	8	THI
Beukes	2022	USA	200	200	PC	iCBT		8	TFI
Kaldo	2013	Sweden	293	293	PC	iCBT		8	TRQ
Kaldo	2008	Sweden	51	26	PC	iCBT	CBT	8	TRQ
				26	PC	iCBT	CBT	8	THI
Jasper	2014	Germany		41	PC	iCBT	CBT	10	THI
				41	PC	iCBT	DF	10	THI
Hesser	2012	Sweden	99	32	PC	iCBT	DF	8	THI
				35	PC	ACT	DF	8	THI
Beukes	2018	UK	146	73	PC	iCBT	DF	8	TFI
Beukes	2018	UK	92	46	PC	iCBT	F2F	8	TFI
									THI
Beukes	2021	USA	27	27	PC	iCBT		8	TFI
Abouzari	2021	USA	30	20	Smartphone	iCBT + sound therapy	DF	8	THI
Beukes	2017	UK	37	37	PC	iCBT + therapist		8	TFI
Malinvaud	2016	France	148	61	PC	Virtual reality	CBT	12	THI

RCT: randomized control trial; iCBT: internet-based cognitive behavioral therapy; CBT: cognitive behavioral therapy; DF: discussion forum; ACT: acceptance and commitment therapy; F2F: face-to-face; THI: Tinnitus Handicap Inventory; TFI: Tinnitus Functional Index; TRQ: Tinnitus Reaction Questionnaire.
